# EPH-ephrin signaling in hyperoxia induced lunginjury

**DOI:** 10.1186/2197-425X-3-S1-A563

**Published:** 2015-10-01

**Authors:** JM Lee, CY Kim, JH Shin, SH Lee, JH Song, MS Park, YS Kim, SK Kim, J Chang, KS Chung

**Affiliations:** Severance Hospital, Yonsei University College of Medicine, Seoul, Republic of Korea

## Introduction

EPH-ephrin interactions have important roles in cell adhesion-based process during inflammation such as the disruption of endothelial-epithelial barriers and adhesion of leukocytes to endothelial cells allowing leukocyte egress into the extracellular space and increasing the leakiness of the endothelial barrier. This is a similar phenomenon with hyeroxia-induced toxicity which is a common complication of critical care practices involving supplemental oxygen therapy. However, little is known about the role of the EPH-ephrin pathway in hyperoxic acute lung injury.

## Objectives

To investigate whether blockade of EPHA2 or EPHB4 using siRNA under hyperoxia can modulate function of human airway epithelial cell line (BEAS-2B). And to explore the time course changes of expression of EPH in lung tissue of mouse exposed to >95% oxygen for 0-72 hr in the hyperoxia-induced mouse lung injury.

## Methods and Results

When BEAS-2B was exposed to hyperoxia (>95%) for 24 hours, Inflammatory markers such as monocyte chemotactic protein 1(MCP-1), chemokine (C-X-C motif) ligand 1 (CXCL2), IL-1b, and IL-6 were more significantly decreased in the group treated with EphA2 siRNA or EphB4 siRNA than the control group. In hyperoxia-induced lung injury mouse model, hyperoxia induced the time dependent increase of alveolar cell count, protein, and lung injury. On western blot of lung lysate, expressions of EphA2, Akt, and BCL-2 increased over time. On the other hand, expression of ERK decreased over time.

## Conclusions

Our preliminary data showed that hyperoxia-induced epithelial injury might be related with EPH-ephrin pathway.

## Grant Acknowledgment

This research was supported by Basic Science Research Program through the National Research Foundation of Korea(NRF) funded by the Ministry of Science, ICT and Future Planning (NRF-2014R1A1A1038278)
